# Distinct chemical composition and enzymatic treatment induced human endothelial cells survival in acellular ovine aortae

**DOI:** 10.1186/s13104-021-05538-3

**Published:** 2021-04-07

**Authors:** Morteza Heidarzadeh, Reza Rahbarghazi, Shirin Saberianpour, Aref Delkhosh, Hassan Amini, Emel Sokullu, Mehdi Hassanpour

**Affiliations:** 1 Stem Cell Research Center, Tabriz University of Medical Sciences, Tabriz, Iran; 2 Department of Applied Cell Sciences, Faculty of Advanced Medical Sciences, Tabriz University of Medical Sciences, Tabriz, Iran; 3 Vascular and Endovascular Surgery Research Center, Mashhad University of Medical Sciences, Mashhad, Iran; 4 Koç University Translational Medicine Research Center (KUTTAM) Rumeli Feneri, Sarıyer, Istanbul, Turkey

**Keywords:** Ovine aorta, Acellularization, Endothelial cells, Survival rate, Attachment

## Abstract

**Objective:**

The current experiment aimed to assess the impact of detergents such as 3% Triton X-100, 1% peracetic acid, 1% Tween-20, and 1% SDS in combination with Trypsin–EDTA on acellularization of ovine aortae after 7 days.

**Results:**

Hematoxylin–Eosin staining showed an appropriate acellularization rate in ovine aortae, indicated by a lack of cell nuclei in the tunica media layer. DAPI staining confirmed the lack of nuclei in the vascular wall after being exposed to the combination of chemical and enzymatic solutions. Verhoeff-Van Gieson staining showed that elastin fibers were diminished in acellular samples compared to the control group while collagen stands were unchanged. CCK-8 survival assay showed enhanced viability in human umbilical vein endothelial cells 5 days after being cultured on decellularized samples compared to the cells cultured on a plastic surface (p < 0.05). SEM imaging showed flattening of endothelial cells on the acellular surface.

## Introduction

Following the onset of chronic macro- and micro-scale vascular pathologies, bypass surgery is highly recommended in most cases [1]. Due to a lack of sufficient vascular tissue grafts, and tissue-matched donors, tissue engineering modalities were exploited to develop appropriate natural and synthetic vascular grafts for transplanting to the target sites [1–4]. Natural vascular grafts have advantages compared to fully synthesized vascular products [5]. In this regard, tissue engineering provides novel methodologies instead of conventional approaches to fabricate an intact vascular system. One of the controversial issues is to design 3D scaffolds for appropriate tissue regeneration with the ability to promote vascularization inside the transplants [1]. Up to the present, numerous authorities tried to use transplants with natural ECM that are comparable to the vascular niche, promoting ECs survival and functional behavior [2]. Notably, natural vascular grafts have a unique 3D microenvironment with the ability to initiate suitable EC alignment and juxtacrine interaction [1, 6]. Also, the existence of ECM proteins in natural vascular graft circumvents the further manipulations required for the preparation of synthetic engineered vascular grafts [7].

In this regard, decellularization is an attempt to exclude host cells and eliminate immunogenic antigens without prominent damage to ECM alignment [8]. In particular, decellularization policies are classified into two main categories which are based on chemical/enzymatic and mechanical approaches [8, 9]. Chemicals/enzymes belong to surfactants can easily diffuse phospholipids of the cell membrane contributing to complete cell lysis. SDS and Triton-X100 are famous surfactants that appropriately eliminate cellular content and genetic materials. However, these agents may contribute to ECM injury. It was stated that Triton-X100, a non-ionic agent, is used for SDS removal with less damaging effect compared to SDS [7–11].

In addition to the use of various materials, mechanical approaches are also applicable to the acellularization of tissues [10, 12]. High mechanical pressure is commonly used with reagents and different temperatures for cells and genetic materials removal [13]. Each method per se has a unique feature and procedure but in all protocols, the washing process of decellularized grafts is integral to appropriately eliminate cell debris and denatured materials [10]. In this regard, continuous freeze-throw cycles could be utilized for increasing decellularization efficiency, however, these approaches are unable to completely remove DNA and other nucleic acid elements, contributing to unwanted immunogenic responses post-transplantation [14, 15]. Although the use of high hypotonic pressure is helpful for efficient cell lysis and removal of genetic material it leads to excessive ECM denaturation and loss of mechanical resistance [16].

Considering different acellularization methods that are applicable for vascular tissues, inherent limitations and disadvantages must not be neglected. Therefore, novel approaches are needed to be developed for successful and high-quality decellularization.

## Main text

### Materials and methods

#### Ovine aorta collection

Sheep aorta was harvested from a local abattoir placed in Tabriz (Northwest of Iran). The samples were kept in ice-cold heparinized PBS and immediately transferred to Stem Cell Research Center (Tabriz University of Medical Science) and kept at − 20˚C until use.

#### Decellularization

Decellularization was performed by using a modified protocol to eradicate various cell types. In brief, a solution containing 3% Triton X-100 (Sigma-Aldrich), 1% peracetic acid (Sigma-Aldrich), 1% Tween-20 (Sigma-Aldrich), 1% SDS (Sigma-Aldrich) was used. After removal of visceral adipose tissue, ovine aortae were washed three times with PBS and incubated in decellularization solution with gentle shaking at room temperature for 7 days. Following the completion of cellular lysis, samples were exposed to 0.05% Trypsin/EDTA (Gibco) solution to obtain high-quality acellular tissue. Thereafter, accellular samples were washed overnight with D.W for chemicals and residual agents.

### Histological examination

#### Hematoxylin–Eosin staining

The existence of cells in the decellularized vessel walls was monitored by histological staining as previously described [17]. For this purpose, decellularized aortae were fixed in 10% formalin solution, 5 µm-thick sections were prepared from paraffin-embedded blocks and stained with H & E solution. Slides were photographed by using an Olympus microscope (Model: BX41; Japan).

#### Immunofluorescence staining

To ascertain the efficiency of our protocol in removing cell nucleus from ECM, we performed immunofluorescence staining by using DAPI. To this end, decellularized samples were submerged in the Tissue Plus Optimal Cutting temperature medium (Scigen; USA) and snap-frozen. Then, 5 µm-size thick sections were prepared by using a Cryostat apparatus (Leica) and placed on poly-L-lysine coated slides. Frozen samples were put in RT temperature washed twice in phosphate-buffered saline solution (each for 5 min). To reduced background staining, we treated the slides with 1% FBS for 20 min. Then, samples were incubated 1 µg/ml DAPI (4′, 6-diamidino-2-phenylindole; Sigma-Aldrich) solution for 30 s.

#### VVG staining

To evaluate the integrity of collagen and elastin fibers, VVG staining was done. In brief, after the preparation of 5 µm-thick sections from ovine aortae, slides were incubated in Verhoeff’s solution for 1 h and washed with tap water. Thereafter, samples were exposed to 2% FeCl_3_ (Sigma-Aldrich) for 5 min followed by one treatment with 5% sodium thiosulfate (Sigma-Aldrich). For counterstaining, slides were stained with VVG’s solution for 5 min. In the next step, samples were dehydrated by using 95% alcohol, and two changes of 100% alcohol. Slides were photographed by using an Olympus microscope (Model: BX41; Japan).

#### Cell culture

HUVECs (National Cell Bank of Iran, Pasteur Institute of Iran, Tehran) were cultured on the decellularized ovine aortae to assess the angiogenesis potential. Samples were sliced into small pieces with 7 mm diameter and 2 mm thickness and placed into each well of 96-well plates. The wells were washed with 70% EtOH (Merck) and sterile D.D.W and repeated several times to remove chemicals used for acellularization. Then, HUVECs (approximately 5 × 10^5^ HUVECs) were re-suspended in 200 µl DMEM/HG (Gibco) containing 10% FBS and 1% Pen-Strep (Gibco) and added to each well and maintained at 37˚C under the humidified condition with 5% CO_2_ for 5 days.

#### Cell survival analysis

To evaluate the cytoprotective effects of the decellularized ovine aorta on the HUVECs viability, we used the Cell Counting Kit-8 assay (Cat no: 96992; Sigma‐Aldrich, Germany) was performed. On day 5 post-HUVECs plating on decellularized samples, we added 10-µl CCK-8 solution to each well and maintained the plates for 3–4 h inside a standard incubator. Then, the optical density of each well was measured at 570 nm by using a microplate reader (ELx808, BioTek). The survival rate was compared to the control HUVECs plated on plastic surface and expressed as % of control.

#### SEM imaging

SEM imaging was performed to assess HUVECs attachment to the ovine aortae after acellularization. Samples were fixed in 2.5% glutaraldehyde (Merck) and snap-frozen by using a freeze-dryer (Operon Co. Ltd., Korea). Thereafter, the surface of the samples was gold-coated and imaged by SEM (Model: MIRA3 FEG-SEM, Tescan).

### Statistical analysis

Data are presented as mean ± SD. We performed a student t-test analysis to find statistically significant differences between the groups. P < 0.05 was considered statistically significant.

### Results

#### Gross and microscopic evaluations

**Fig. 1:**
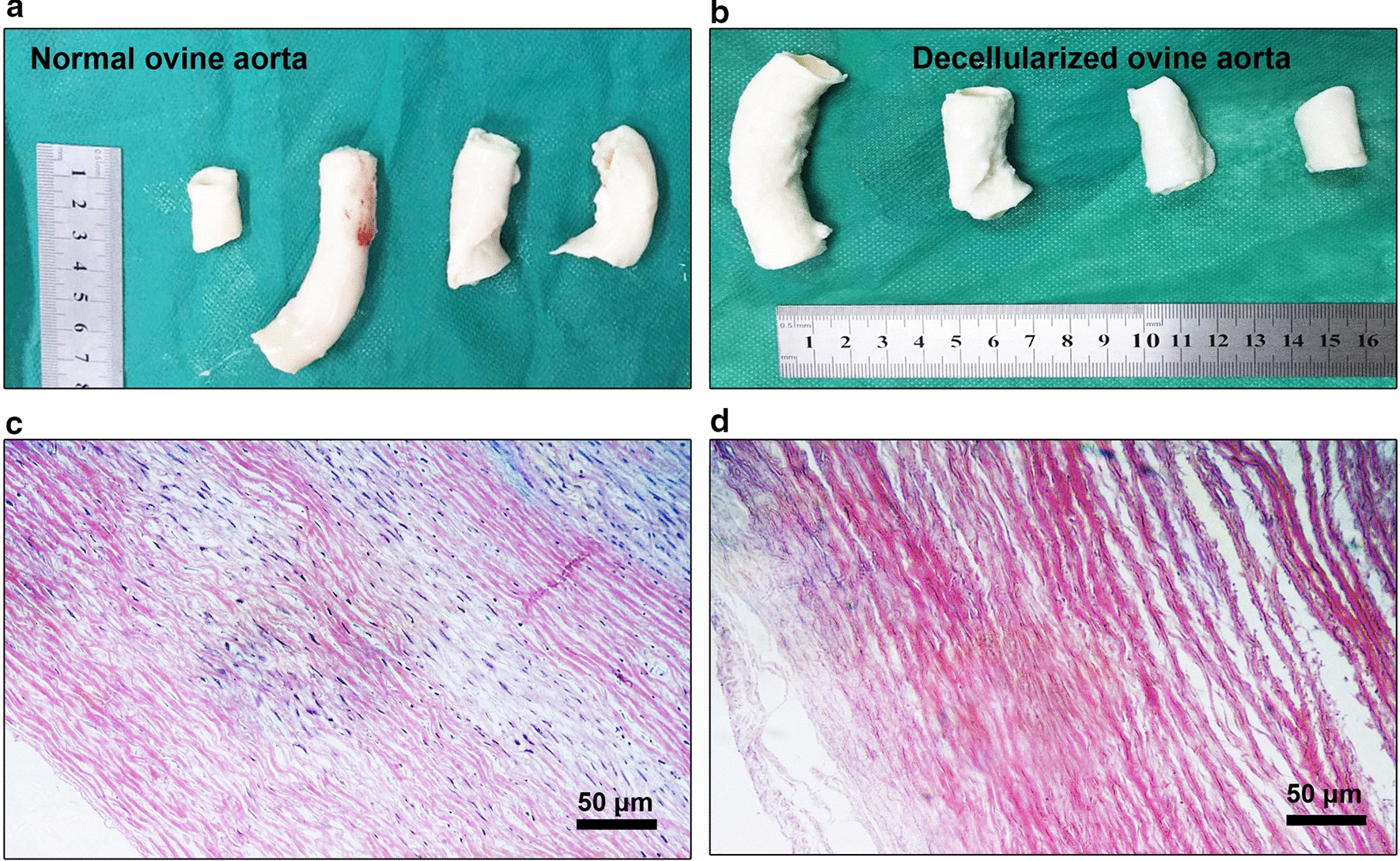
The appearance of ovine aortae pre- and post-decellularization of day 7 (**a**, **b**). Hematoxylin–Eosin staining revealed a large number of cell nuclei in the tunica media layer while 7-day incubation of ovine aortae with decellularization solution and enzymatic treatment eliminated cell nuclei (**c**, **d**)

In a gross examination, the strength and elasticity of decellularized scaffolds were comparable to the original ovine aortae with a little more softness (Fig. 1a, b). It is logical to imagine that the cells and small-sized ECM components removal contributed to minor consistency reduction upon treatment with acellularization solution. H & E staining showed the presence of aligned smooth muscle cells nuclei in normal ovine aortae (Fig. 1c, d). The use of acellularization protocol eliminated cellular components in which eccentric tunica media fibers were devoid of nuclei. It's important to say that the distance of tunica media fibers increased post-decellularization. One reason would be that the parallel connection of fibers would decrease during consecutive washes with an acellularization buffer. Meanwhile, it is noteworthy to state that the treatment of enzymatic solution, Trypsin–EDTA, facilitates small-size fibers such as elastin.

**Fig. 2:**
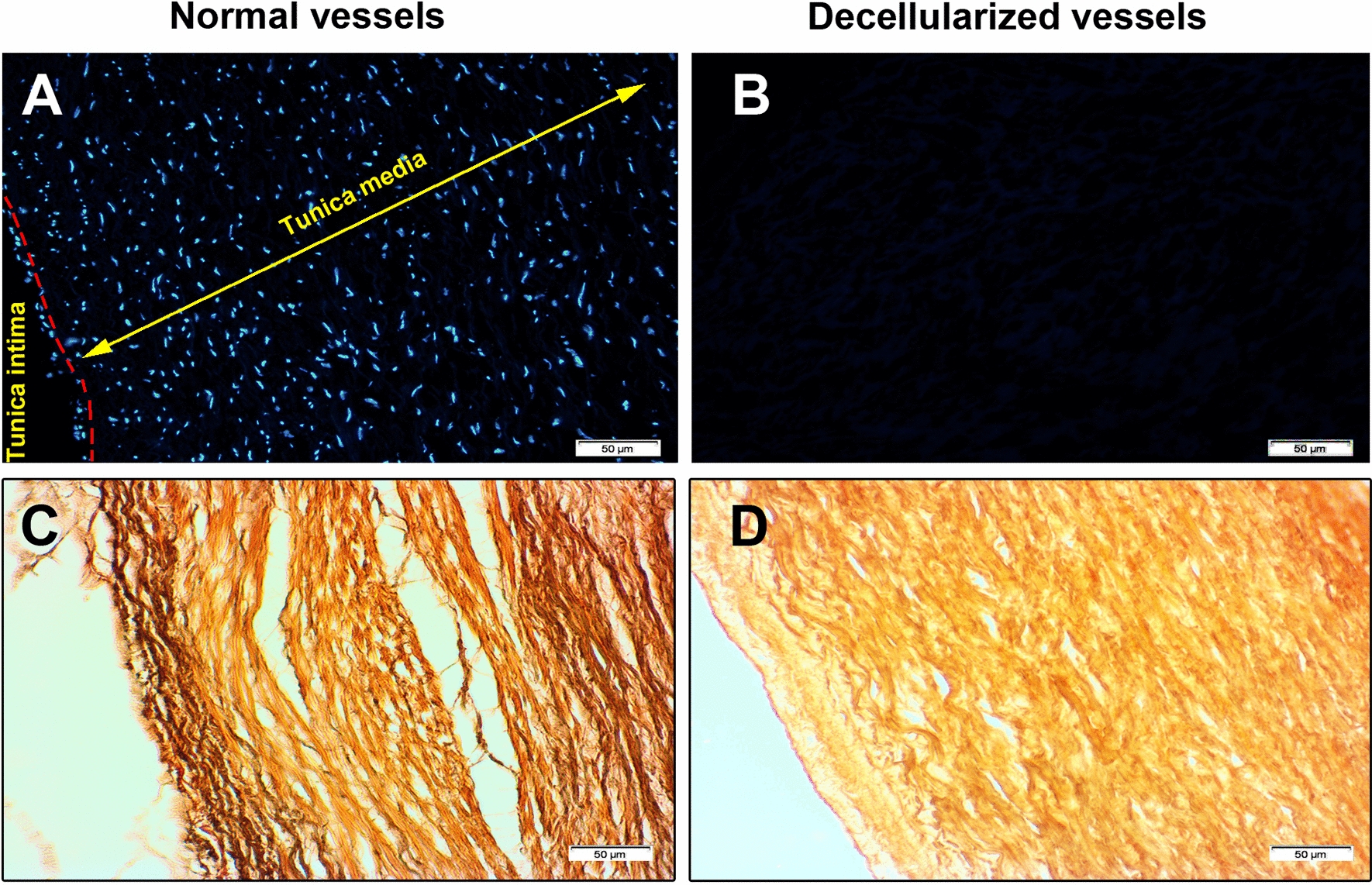
Immunofluorescence imaging of decellularized ovine aortae with DAPI (**a**, **b**). 7-day incubation of samples with decellularization buffer and enzymatic treatment (**b**) yielded in cell nuclei removal from vascular intima and media layer compared to the control sample (**a**). Verhoeff-Van Gieson staining revealed a decrease of elastin fibers (black strands) in decellularized samples while levels of collagen fibers (yellow fibers) remained unchanged (**c**, **d**)

To ascertain, removal of nuclei from ovine aortae, we performed immunofluorescence imaging DAPI appropriate for detection of nuclear genome (Fig. 2a, b). As expected, data confirmed the presence of multiple nuclei in the vascular media and intima layer in normal ovine samples (Fig. 2a). Treatment of ovine aortae with detergents and Trypsin resulted in the elimination of nuclei and genome, indicating an effective cell removal (Fig. 2b). Nuclear remnants along with other cellular components are touted as the main reasons for transplant rejection [18].

By using VVG staining, we monitored tissue levels of elastin and collagen fibers pre- and post-acellularization (Fig. 2c, d). Bright-field microscopic images showed the presence of black color elastic fibers in a meshwork of collagen stands colored yellow. Seven-day incubation with detergents and Trypsin solution contributed to the decrease of elastin fibers while a yellow color background was observed post-acellularization (Fig. 2c, d). These data showed the use of Triton X-100, peracetic acid, Tween-20, and SDS along with enzymatic solution decreased the elastin fibers in vascular structure while levels of collagen fibers were unchanged.

#### Decellularized ovine aortae promoted human endothelial cells survival

**Fig. 3:**
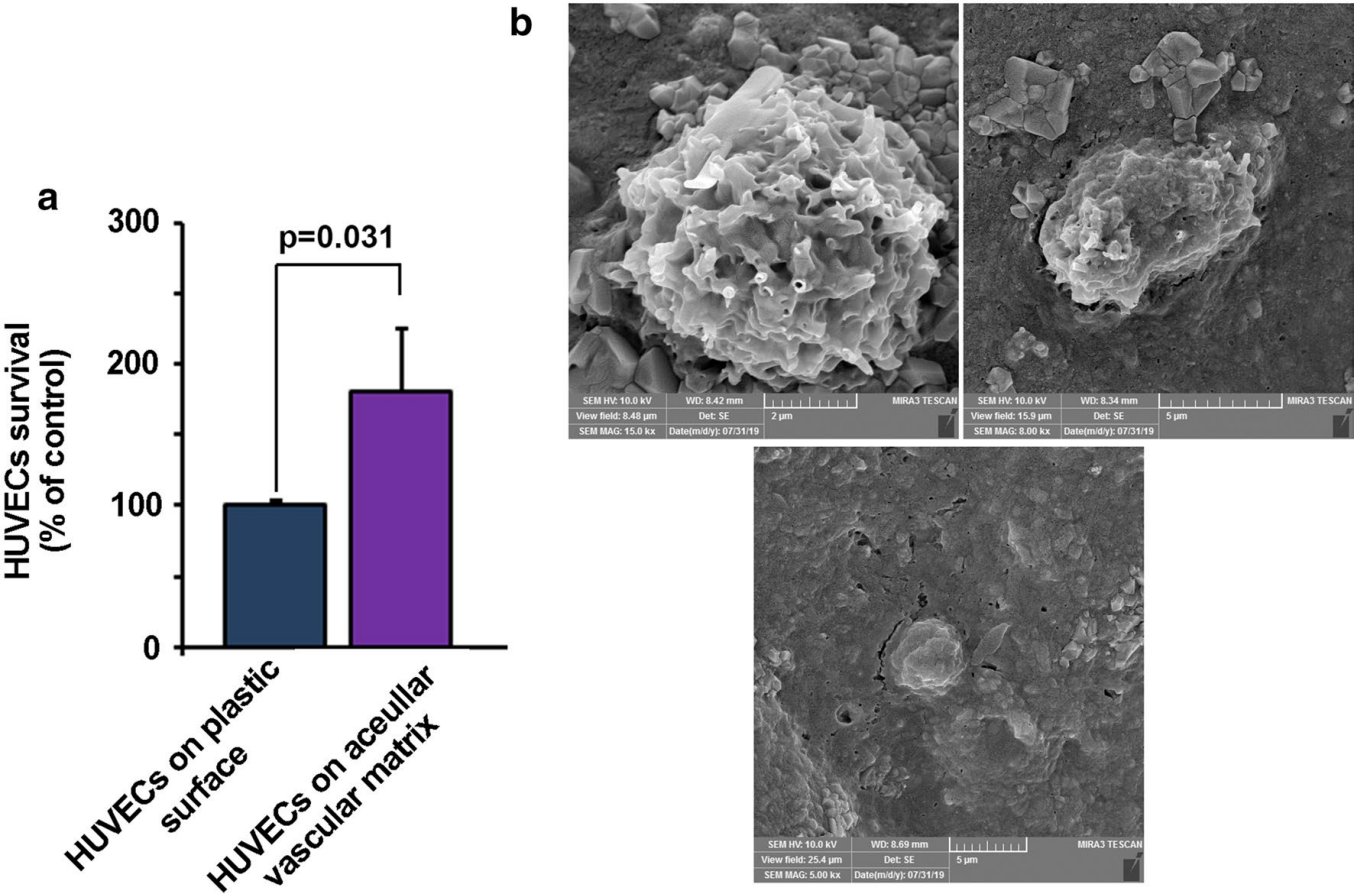
CCK-8 assay showed an increased HUVECs survival 5 days after being plated on the acellular aortae (n = 5; p < 0.05; **a**). SEM imaging of HUVECs plated on the acellular surface showed cell attachment (**b**) and growth (**c**) after 5 days

To evaluate the efficiency of ECM substrates after treatment with acellularization protocol, we performed a CCK-8 assay. Data showed that the 5-day culture of the HUVECs on decellularized ovine aortae increased survival rate compared to the control cells expanded on the plastic substrate (p < 0.05; Fig. 3a). Based on data from the CCK-8 panel, we found an approximate 80% in cell survival rate compared to the control HUVECs (Fig. 3a). These data demonstrated that our acellularization protocol maintained the capacity of ECM substrates to promote the viability of human endothelial lineage.

#### SEM imaging revealed cell attachment to decellularized aortae

Data from SEM analysis confirmed the attachment of HUVECs to decellularized ovine aortae. According to our data, HUVECs covered the surface of decellularized ovine aortae 5-day after being-plated (Fig. 3b). Imaging showed that cells were easily distributed on the decellularized matrix. These data showed the potency of ECM substrates and the existence of motifs to maintain HUVECs-to-ECM connection after acellularization.

### Discussion

This study was performed to investigate the regenerative potency of acellular ovine aortae treated with the combination of detergents and Trypsin solution. Our data confirmed the cell lysis and removal after 7-day incubation of ovine aortae with acellularization protocol. By using H & E staining and immunofluorescence imaging, we confirmed the absence of nuclei, especially in the vascular tunica media inner and outer layers. Therefore, the simultaneous application of enzymatic and multiple detergents caused the proper permeability of chemical to deep parts of tunica media. A slight decrease in vascular consistency could be correlated with small-sized fibers removal, mainly elastin, and interruption of parallel cross-links between the ECM substrates [19]. We also found that the 5-day culture of HUVECs on acellular ECM contributed to an increased cell survival rate. The maintenance of micro and nanofibrous structures like vascular tissue, in the levels of single molecules and connective tissue fibrils and fibers, could help endothelial cells to attach efficiently to the beneath surface [20]. The current protocols with specific components and periods were found to be eligible for the promotion of HUVECs survival and expansion. Commensurate with these comments, it is rational to imagine that the existence of motifs and 3D folds at molecular levels should be considered for each tissue type submitted to the acellularization to reduce detrimental effects of chemicals [8, 21]. The attachment of human endothelial cells to the acellular surface was evident in SEM images. The cells acquired potency to proliferate and covered vascular luminal surface. The existence of specific molecules mainly glycosaminoglycans is conceived as one of the most important factors of tissue rejection after transplantation [22]. Although, these factors could facilitate cell attachment and activate specific signaling pathways correlated with cell growth and survival [23]. This study did not monitor the existence and possibility of glycosaminoglycans in acellular ovine aortae. Besides, we did not perform multiple tissue engineering analyses to assure mechanical consistency and resistance to pressures.

### Conclusions

In in study, we demonstrated that the use of both enzymatic cocktails with chemical composition could be an alternative method to prepare the acellular vascular structure to culture and expand human ECs.

## Limitations

In the current experiment, we showed the efficiency of a chemical cocktail in combination with an enzymatic solution to prepare decellularized aortic grafts. The sample size of the study is limited. Future experiments are required to address the cell-to-cell connection and which adhesion molecules such as VE-Cadherin are expressed by the cells after being cultured on the decellularized ovine aortae.

### Implications

Development of engineered vascular grafts could help human medicine to accelerate regeneration in the injured tissues by providing blood supply. Due to the small number of volunteers and the possibility of disease transmission, the use of non-human vascular grafts is mandatory. In the current experiment, we prepared a decellularized vascular graft from ovine aorta using chemical and enzymatic approaches that provided a suitable niche for the attachment and growth of human endothelial cells.

## Data Availability

All data generated or analyzed during this study are included in this published article.

## Abbreviations

D.W: Distilled water
D.D.W: Double-distilled water
ECs: Endothelial cells
ECM: Extracellular matrix
FBS: Fetal bone serum
H & E: Hematoxylin–Eosin
DMEM/HG: High content glucose Dulbecco's Modified Eagle Medium
HUVECs: Human Umbilical Vein Endothelial Cells
Pen-Strep: Penicillin–Streptomycin
PBS: Phosphate buffer saline
RT: Room temperature
SEM: Scanning electron microscopy
SDS: Sodium dodecyl sulfate
3D: Three dimensional
VVG: Verhoeff-Van Gieson
